# Improving Cardiopulmonary Resuscitation Quality and Resuscitation Training by Using Real-Time Audiovisual-Assisted Devices

**DOI:** 10.7759/cureus.68757

**Published:** 2024-09-05

**Authors:** Jerin Varghese, Abin Thomas, Bharath S Prasad, Sreekrishnan Trikkur, Sabarish Nair, Gireesh Kumar, Naveen Mohan, Manna M Theresa

**Affiliations:** 1 Department of Emergency Medicine, Amrita Institute of Medical Sciences, Kochi, IND

**Keywords:** audiovisual devices, cardiac arrest, cpr, feedback devices, non medical care providers

## Abstract

Context

Cardiac arrest occurring outside of a medical facility is a significant cause of death and disability worldwide. In developing nations, it accounts for a notable portion of total mortality, with only a small percentage of those affected surviving beyond the initial emergency department intervention. Despite the critical importance of high-quality cardiopulmonary resuscitation (CPR) in these situations, there has been limited research on the effectiveness of audiovisual feedback devices in improving CPR performance among laypersons or non-medical caregivers. These caregivers, often untrained in advanced medical procedures, play a crucial role in the immediate response to cardiac arrest before professional help arrives. This study aims to address this gap by evaluating the impact of such devices on CPR quality delivered by non-medical individuals.

Aim

This study aimed to determine whether the use of audiovisual devices would improve cardiopulmonary resuscitation performance among non-medical care providers.

Materials and methods

Using a multiple-choice questionnaire, an audiovisual aid-based prospective observational study (non-interventional observation study) was conducted at a medical college hospital in Kochi from June 2022 to February 2024. A minimum sample size of 66 was derived from pilot studies, with 95% confidence and 90% power. A total of 146 participants met the inclusion criteria (non-medical personnel of 18-50 years of age). After the exclusion of pregnant women and non-interested participants, the study participants were analyzed for the quality of cardiopulmonary resuscitation in a mannequin with the help of audiovisual devices. Statistical analysis was conducted using IBM SPSS Statistics for Windows, Version 20 (IBM Corp., Armonk, New York, released in 2011). Categorical variables were expressed as frequency and percentage. McNemar's Chi-square test was applied to compare the CPR compression rates with and without the visual feedback device, and the kappa statistic was used to assess how consistently participants performed within the same compression rate category (less than 100, 100-120, or more than 120 compressions per minute) with and without the feedback device.

Results

The improvement in CPR quality, which was visualized as a green color in the CPR feedback device, was significant, with 109 participants (74.7%) showing good outcomes. The chest compression rate also significantly improved from 95 to 117 with the use of feedback devices (p -0.011, Kappa - 0.167) among non-medical care providers. While the kappa value suggests that some variability exists in compression rates when switching between with and without feedback, the overall improvement is still noteworthy.

Conclusion

The majority of participants (74.7%) were able to consistently maintain green light in the visual feedback watch, which means their chest compression rate was within optimal range (100-120/min) when using the audiovisual feedback device. This indicated that use of audiovisual devices significantly improved compression rates among non-medical care providers and effectively helped them perform high-quality CPR.

## Introduction

Cardiac arrest, which is characterized by the cessation of cardiac activity, presents a prevalent public health challenge [1,2]. When it occurs outside of a medical facility, it stands as a prominent cause of death and disability globally, constituting 10% of general mortality in developing nations [3]. The mortality rate of patients experiencing cardiac arrest is high, with only a small percentage surviving beyond the emergency department [4].

The treatment approach for cardiac arrest aims to achieve the return of spontaneous circulation (ROSC) by adhering to the chain of survival, which encompasses activating emergency response, promptly starting basic cardiopulmonary resuscitation (CPR), early defibrillation, initiating advanced resuscitation, commencing post-cardiac arrest care, and facilitating recovery. Any weakness in these steps diminishes the likelihood of survival in cases of out-of-hospital cardiac arrest [2,5].

The concept of CPR was introduced and documented by Peter Safar in 1960 [4], with the first CPR guidelines established by the American Heart Association (AHA) in 1966 [5]. Since then, CPR techniques have undergone refinement based on evidence-based recommendations from international resuscitation councils such as the International Liaison Committee on Resuscitation (ILCOR), AHA, and the European Resuscitation Council (ERC).

Following its documentation, CPR gained widespread adoption due to its feasibility for performance by anyone, including laypersons, without the need for surgical instruments. CPR, along with advanced cardiac life support, has remained the standard method of resuscitation since it was introduced [6]. In essence, CPR involves attempting to restore spontaneous circulation through chest compressions, with or without ventilation [1,7,8].

The early interventions implemented through the chain of survival have led to gradual but steady improvements in survival rates [3]. These rates vary significantly and are influenced by numerous factors such as age, parental education level, season, exercise status, location of the patient during the arrest, time taken to initiate CPR, underlying medical conditions of the patient, initial electrocardiogram (ECG) rhythm, level of knowledge and skills, presence of an emergency medical service (EMS) system, early defibrillation, hemodynamic changes, blood loss, and other variables [7-10]. Enhancing survival outcomes from cardiac arrest remains a primary objective for emergency medical teams globally, given the annual incidence of over six million cardiac arrests worldwide. Efforts persist in identifying the essential components of resuscitation to enhance survival rates further.

The delivery of chest compressions constitutes a crucial aspect of cardiopulmonary resuscitation (CPR) [9,10]. AHA currently advises reducing interruptions in chest compressions to maximize the number of compressions delivered per minute [10].

Chest compression fraction (CCF) is the measure of the total time spent on chest compressions during a resuscitation attempt, divided by the total duration of the event [11]. For example, if CPR is being delivered over a two-minute interval, and compressions are delivered for one and a half minutes during that time, the CCF would be 75%. The formula to calculate CCF is "Total time of cardiac arrest event" divided by "Total time of chest compressions" × 100%. It serves as an indicator of the time dedicated exclusively to chest compressions. CCF is critical for survival rates because higher fractions are associated with better outcomes during cardiac arrest. Research indicates that a CCF of at least 60-80% is optimal, meaning that rescuers should aim to minimize interruptions to chest compressions as much as possible. This uninterrupted delivery of compressions ensures that blood flow to vital organs, especially the brain and heart, is maintained, which is essential for increasing the likelihood of survival and reducing the risk of neurological damage. Rescuers should maintain a chest compression rate of 100 to 120 per minute and a depth of at least 2 inches, while avoiding depths greater than 2.4 inches or 6 centimeters, in accordance with the latest AHA guidelines [11]. Setting a target chest compression fraction of at least 60% is intended to minimize interruptions in compressions, thereby maximizing blood flow and coronary perfusion during CPR. Calculating the CCF provides a means to assess and enhance the quality of future resuscitation efforts [11].

During resuscitation, providers might intermittently stop chest compressions for critical patient care tasks, including rhythm and pulse checks, airway management, ventilation, or defibrillation. However, interruptions in compressions can also happen due to factors not directly related to patient care, such as distraction, fatigue, confusion, or a lack of proficiency in executing resuscitation techniques effectively.

Interruptions in chest compressions during CPR are known to adversely affect cerebral and coronary perfusion, as evidenced by animal studies on cardiac resuscitation [7]. Furthermore, the quality of chest compressions by rescuers deteriorates rapidly over time due to improper compression rate and depth, rescuer fatigue, and distractions [10]. CPR feedback devices are specifically designed to address these issues mentioned above [11-13] and ensure that the quality of compressions remains within the optimal range by following mechanisms:

1. Reducing Interruptions and Maintaining Chest Compression Fraction (CCF) Interruptions during chest compressions can occur for various reasons, including rescuer transitions, equipment adjustments, or simply due to distractions. These interruptions can significantly lower CCF, thereby reducing the effectiveness of CPR. Feedback devices help in the following ways: (i) continuous monitoring, auditory and visual cues (emit beeps or display lights to signal the need to continue compressions, which helps to reduce unnecessary pauses and distractions) and (ii) Guidance for timing transitions: feedback devices can help manage transitions between rescuers or the application of defibrillation, ensuring that interruptions are brief and that compressions are resumed promptly.

2. Ensuring Optimal Compression Rate and Depth CPR feedback devices address this by (i) real-time rate monitoring: these devices track the rate of compressions and provide immediate feedback if the rate falls below or exceeds the recommended range and (ii) depth feedback: the devices measure the depth of each compression and alert the rescuer if they are compressing too shallowly or too deeply.

3. Combating Rescuer Fatigue Rescuer fatigue is a significant factor that can lead to a decline in CPR quality over time. Fatigue often results in shallower compressions and a slower rate as the rescuer tires. CPR feedback devices help to mitigate these effects by generating objective performance data and immediate alerts: Rescuers may not always realize when fatigue is affecting their performance. Feedback devices provide objective data and alerts that can prompt a switch before the quality of compressions drops below acceptable levels.

4. Reducing Distractions In high-stress situations like cardiac arrest, distractions are common and can lead to poor CPR performance. Feedback devices help keep rescuers focused by making use of simplified decision-making and consistent cues: With real-time feedback, rescuers do not have to constantly think about whether they are performing CPR correctly. The device guides them, allowing them to focus on other critical aspects of the resuscitation process and provide consistent cues (like beeping for the correct rate) that help rescuers stay on track, even in chaotic environments.

Existing evidence highlights the benefits of audiovisual feedback devices in CPR training, particularly in improving the quality of chest compressions by providing real-time corrective feedback on compression rate, depth, and recoil. However, research specifically focusing on the use of these devices among non-medical caregivers is limited. Studies have shown mixed results regarding the effectiveness of standalone audiovisual feedback devices in layperson CPR training, indicating a need for further investigation.

This gap in research underscores the importance of studying the impact of real-time audiovisual-assisted devices on CPR quality and training among non-medical caregivers. Such a study could provide valuable insights into how these devices can enhance the effectiveness of CPR performed by non-professionals, ultimately improving patient outcomes.

This study aims to determine whether the use of audiovisual devices would improve cardiopulmonary resuscitation performance among non-medical care providers.

## Materials and methods

Study design

A questionnaire was administered and an audiovisual aid-based prospective observational study was done on 146 participants. This study involves observing and collecting data on participants over a specified period to assess the impact of the use of audiovisual feedback devices during CPR performed by non-medical caregivers. As an observational study, the researchers did not actively manipulate any variables beyond introducing the feedback devices. The study did not involve random assignment to different interventions; rather, it observed the natural use of audiovisual feedback devices during CPR to evaluate their effectiveness. The data were collected based on participants' performance of CPR both with and without the use of these devices. This provided a comparison within the same group of participants, making it possible to observe how the feedback devices influenced their performance over time.

There was no external group of participants who were observed performing CPR without any intervention throughout the study. The focus was entirely on the effect of adding the audiovisual feedback devices to the same individuals who initially performed CPR without them. Each participant served as their own control, as their CPR performance without the feedback devices was compared to their performance with the devices. This method is effective in reducing variability caused by individual differences since each participant's baseline performance serves as the reference point.

Study setting

The study was conducted between June 2022 and February 2024 at the emergency medicine department and emergency intensive care unit (ICU) of an Indian medical college hospital with an annual emergency department patient load of around 50,000 as there was an associated wide classroom where the study could be conducted easily.

Sample size

A pilot study was conducted with 10 samples. With an effect size of 0.406, 90% power, and 95% confidence, the minimum sample size was determined to be 66. The mean compression rate with a feedback device was 16.3 compressions per minute (±2.62) and the mean compression rate without the feedback device was 15.2 compressions per minute (±2.29). The final sample size was 146 participants because a large number of participants were present. So data were collected from them and was included in the study. The sample size was not recalculated or adjusted based on the pilot study results.

Data sources/measurement

The inclusion criteria were participants aged between 18 and 50 years, coming to the emergency department for the study who were non-medical personnel. This age range is chosen to ensure that participants are physically capable of performing CPR effectively, as younger and older individuals might have physical limitations that could impact their ability to perform chest compressions. The exclusion criteria include pregnant women and non-interested participants. Pregnant women were excluded to avoid any potential risks to both the mother and the fetus during the physical exertion involved in performing CPR. The physical demands of performing CPR, such as the need for adequate chest compressions, could pose a risk to pregnant women. Non-interested participants refer to individuals who, despite meeting the inclusion criteria (age 18-50, non-medical personnel, coming to the emergency department), explicitly declined to participate in the study and this was due to personal beliefs, lack of perceived benefit, time constraints, experiences, or concerns that make them hesitant to participate in CPR training, like potential physical limitations or ethical considerations.

The study tools included a CPR quality assessing watch (Practi-CRM CPR Wrist Monitor/Watch, WNL Products, Holliston, MA, USA) [14], a questionnaire [15,16], a laptop for audiovisual aid, and a mannequin. The Practi-CRM (WLCRM) fulfills the requirement for visual feedback of compression rate as defined by the AHA: “Device with screen display that detects the rate of actual compressions and indicates visually whether to push faster or to push slower in real-time". As the user performs CPR compressions, the wristband detects the upward and downward movements of the hand. These movements correspond to the compressions being performed. The monitor calculates the compression rate by measuring the time interval between each upward and downward movement.

The study followed a systematic approach to evaluate the impact of audiovisual feedback devices on the quality of CPR performed by non-medical caregivers. Participants received a comprehensive briefing on CPR, covering essential aspects such as recognizing cardiac arrest, the importance of timely CPR, and the correct technique, including chest compression rate, depth, and hand positioning. The briefing was supported by visual aids, including short instructional videos that demonstrated proper CPR technique. Before the practical session, participants completed a pre-test questionnaire designed to assess their baseline knowledge of basic life support (BLS) techniques.

Participants were instructed to perform CPR on a mannequin without the aid of any feedback devices. The focus was on chest compressions, as these are critical to effective CPR. The quality of CPR, specifically the compression rate, was recorded in real time manually during this session. The collected data served as a control to compare against later performance when feedback devices were introduced. Participants were then introduced to the audiovisual feedback device - a CPR quality assessing watch. This device provided real-time feedback on their CPR performance. The watch displayed a green light when compressions were within the recommended range (100-120 compressions per minute) and a red light if compressions were too slow or too fast. Their performance was monitored and recorded in the same manner as during the initial session, along with the data of red and green light displayed during compression. Post-session analysis was also done and involved comparing the pre- and post-intervention CPR quality metrics.

Statistical analysis

Statistical analysis was conducted using IBM SPSS Statistics for Windows, Version 20 (2011 release; IBM Corp., Armonk, New York). Categorical variables were presented as frequency and percentage. McNemar's chi-square test was utilized to determine the statistical significance of differences in compression rates with and without the use of a CPR quality assessment watch and kappa statistic was computed for measuring the agreement. McNemar's chi-square test is particularly suited for analyzing paired nominal data. In this study, participants' CPR performance was measured both with and without the use of an audiovisual feedback device, creating a paired scenario. Since the study outcomes were binary (e.g., "good" or "poor" based on compression rate), McNemar's test was appropriate for determining if the feedback device significantly influenced the shift in these outcomes. The kappa statistic was employed to measure the degree of agreement between the participants' CPR performance outcomes with and without the feedback device. This test is particularly useful for evaluating the consistency or reliability of categorical variables in paired data. Categorical variables, such as the binary outcomes ("good" or "poor" CPR performance), were presented as frequencies and percentages in the study and no continuous variables were analyzed. Participants with missing data were excluded from the analysis, ensuring that only complete data sets contribute to the final results.

## Results

Descriptive data

Among 146 subjects, the majority of the participants (109, 74.7%) demonstrated a "good" outcome and 37 (25.3%) participants had an inadequate outcome (red color). "Good" outcome corresponds to the device displaying a green light consistently during chest compressions. The green light indicates that the participant's chest compression rate was within the optimal range of 100-120 compressions per minute, which is the recommended rate for effective CPR according to current guidelines. This outcome suggests that the participant was able to perform CPR at a rate that maximizes the chances of survival in a cardiac arrest scenario, as it aligns with the best practices for maintaining adequate blood flow during resuscitation A "poor" outcome is associated with the device showing a red light consistently or red and green lights being shown intermittently during chest compressions. The red light indicates that the compression rate was either below 100 compressions per minute or above 120 compressions per minute, both of which are considered suboptimal (Table 1).

**Table 1:** Distribution of outcome

Outcome	Number of participants	Percentage (%)
Good (Green color)	109	74.7
Poor (Red color)	37	25.3

The use of the visual feedback device resulted in a significant improvement in the number of participants achieving the optimal compression rate of 100-120 compressions per minute, increasing from 65.1% without the device to 80.1% with the device. Additionally, the percentage of participants performing compressions at a suboptimal rate (<100 compressions/min) decreased from 28.8% to 17.1%, which suggests that the device successfully alerts users when their performance is suboptimal, allowing them to adjust in real time (Table 2).

**Table 2:** Distribution of compression rate with respect to the visual feedback device

Compression Rate	With the visual feedback device		Without the visual feedback device	
	Frequency	Percentage (%)	Frequency	Percentage (%)
<100/minute	25	17.1	42	28.8
100-120/minute	117	80.1	95	65.1
>120/minute	4	2.7	9	6.2

The results show a statistically significant improvement in compression rates when using the visual feedback device (p=0.011). The kappa value of 0.167, although indicating a slight agreement, still supports the effectiveness of the feedback device in guiding participants towards the optimal compression rate. The significant improvement in compression rates suggests that the feedback device is particularly effective in correcting rates that are too slow or too fast. The device's ability to provide visual cues allows participants to make necessary adjustments in real time, which is crucial for maintaining the quality of CPR.

The chi-square test and its associated p-value are essential tools in this study for evaluating whether the introduction of the feedback device had a statistically significant impact on the quality of chest compressions, specifically the compression rates. McNemar's chi-square test is particularly suited for analyzing paired nominal data. In this study, participants' CPR performance was measured both with and without the use of an audiovisual feedback device, creating a paired scenario. Since the study outcomes were binary (e.g., "good" or "poor" based on compression rate), McNemar's test was appropriate for determining if the feedback device significantly influenced the shift in these outcomes. The chi-square value of 11.09 represents the degree to which the observed compression rates (with and without the feedback device) deviate from what would be expected if the feedback device had no effect. In this context, the value suggests that there is a noticeable difference in compression rates when participants use the feedback device, as compared to when they do not (Table 3).

**Table 3:** Comparison of compression rate with respect to visual feedback device Chi-square value = 11.09; n=number of participants

Compression without visual feedback device (total subjects)	Compression rate with visual feedback device			p	kappa
	< 100/minute, n (%)	100-120/minute, n (%)	>120/minute, n (%)		
<100/ minute (42)	11 (7.5)	30 (20.5)	1 (7)	0.011	0.167
100-120/minute (95)	11 (7.5)	82 (56.2)	2 (1.4)		
>120/minute (9)	3 (2.1)	5 (3.4)	1 (7)		

The data shows that participants who achieved a good outcome were significantly more likely to maintain or improve their compression rate with the use of the feedback device (p<0.001). The kappa statistic measures the level of agreement between two sets of categorical data; in this case, the consistency of compression rates falling within the optimal range (100-120 compressions per minute) with and without the feedback device. Kappa values typically range from -1 to 1, where 1 indicates perfect agreement and 0 indicates no agreement beyond chance. Negative values indicate disagreement. A kappa value of 0.111, although statistically significant, is considered low and indicates only a slight agreement beyond what would be expected by chance. This suggests that while the feedback device did help some participants maintain optimal compression rates, there was still considerable variability in how well different participants responded to the feedback. The low kappa value might be due to factors such as individual differences in interpreting the feedback, variations in physical ability, or differences in initial CPR knowledge (Table 4). 

**Table 4:** Good outcome (green color) Chi-square value = 20.36; n=number of participants

Compression rate without visual feedback device (total subjects)	Compression rate with visual feedback device			p	kappa
	< 100/minute, n (%)	100-120/minute, n (%)	>120/minute, n (%)		
<100/minute (32)	5(4.6)	27(24.8)	0(0)	<0.001	0.111
100-120/minute (69)	6(5.5)	63(57.8)	0(0)		
>120/minute (8)	2(1.8)	5(4.6)	1(9)		

For participants who displayed poor outcomes (as indicated by the red light), a p-value of 0.475 indicates that the differences in the compression rates for this subgroup (with and without the feedback device) are not statistically significant. This suggests that the feedback device did not have a meaningful impact on improving the compression rates for participants who initially performed poorly. This lack of effectiveness could be due to several factors, such as difficulty in understanding the feedback, physical limitations, or stress, which may hinder these participants from adjusting their performance effectively. The kappa value of 0.341 indicates a fair level of agreement, meaning there was some consistency in the compression rates within the optimal range with and without the feedback device, but not enough to be considered a strong effect. This fair agreement suggests that while some participants in this subgroup did respond to the feedback device, many did not, indicating variability in the device's utility for improving performance in those who initially struggled with CPR (Table 5).

**Table 5:** Poor outcome (red color) Chi-square value = 2.5; n=number of participants

Compression rate without visual feedback device (total subjects)	Compression rate with visual feedback device			p	kappa
	< 100/minute, n (%)	100-120/minute, n (%)	>120/minute, n (%)		
<100/minute (10)	6 (16.2)	3 (8.1)	1 (2.7)	0.475	0.341
100-120/minute (26)	5 (13.5)	19 (51.4)	2 (5.40		
>120/minute (1)	1 (2.7)	0 (0)	0 (0)		

## Discussion

The results indicated that among the 146 subjects, 109 (74.7%) showed a good outcome, while 37 (25.3%) displayed a poor outcome. Further analysis revealed that the use of the audiovisual feedback device resulted in an improvement in compression rate, with 117 subjects (80.1%) achieving the desired compression rate compared to 95 subjects (65%) without the device. This finding is in line with a study by Lin et al. [17], which showed that using real-time feedback devices greatly enhanced the quality of chest compressions performed by rescuers. The study also showed that a higher percentage of participants (from 49.82% to 71.23%) met the target compression rate. Our results align with the existing literature, which highlights that real-time feedback can enhance both the learning process and the practical application of CPR skills. For instance, research by Nishiyama et al. [18], demonstrated that real-time visual feedback improved chest compression quality during CPR training, leading to better overall resuscitation outcomes in clinical settings.

When comparing compression rates with respect to watch usage and outcome, there was a significant difference in compression rates for both good and poor outcomes when using the watch. For participants with a good outcome, compression rates significantly improved with the use of the watch (p-value < 0.001, kappa value = 0.111). However, for participants who had poor outcomes (as indicated by the red light), a p-value of 0.475 indicates that the differences in compression rates for this subgroup (with and without the feedback device) are not statistically significant. This suggests that the feedback device did not have a meaningful impact on improving compression rates for participants who initially performed poorly. This lack of effectiveness could be due to several factors, such as difficulty in understanding the feedback, physical limitations, or stress, which may hinder these participants from adjusting their performance effectively. The kappa value of 0.341 indicates a fair level of agreement, meaning there was some consistency in compression rates within the optimal range with and without the feedback device, but not enough to be considered a strong effect. This fair agreement suggests that while some participants in this subgroup did respond to the feedback device, many did not, indicating variability in the device's utility for improving performance in those who initially struggled with CPR.

The statistical significance (p = 0.011) observed in this study is reinforced by other research. A study by Gugelmin-Almeida et al. [19] reported similar results, indicating significant improvements in compression quality with feedback devices, and highlighted that these devices help in maintaining the recommended compression rate and depth. A study by Yeung et al. [20] found that participants who received feedback on their compression depth and rate during training sessions performed significantly better in subsequent CPR tests than those who did not receive such feedback, suggesting that feedback devices can be an effective tool in bridging the gap between CPR training and real-life application.

While this study focused on immediate performance, similar studies have examined long-term retention. A study by Akizuki et al. [21], indicated that the participants who trained with feedback devices retained a higher quality of CPR skills over a six-month period compared to those who trained without such devices. This suggests that the benefits observed in this study might extend beyond the short term, though further research is needed to confirm this observation.

The inclusion of non-medical personnel in CPR training is crucial, given that bystanders often serve as the first responders in out-of-hospital cardiac arrest (OHCA) situations. AHA emphasizes the importance of high-quality CPR in its guidelines, stating that optimal chest compression rates and depths are vital to increasing the chances of survival. However, non-medical personnel may not have the same level of training or confidence as healthcare professionals. The use of audiovisual aids can help mitigate this gap by providing immediate, clear feedback, thus enhancing the quality of CPR performed by lay rescuers [20,22]. This study contributes to the growing body of evidence that supports the use of such devices in training non-medical personnel, potentially leading to better preparedness and improved outcomes in emergency scenarios.

The findings from this study suggest that audiovisual feedback devices can significantly improve the quality of chest compressions during CPR. However, previous studies have yielded mixed results, which may be attributed to several factors:

1. The effectiveness of feedback devices in improving CPR performance can vary significantly based on the technology used. Earlier studies might have employed less sophisticated devices with limited real-time feedback capabilities or less intuitive interfaces. In contrast, the feedback device used in this study provided immediate visual cues, which likely contributed to its effectiveness.

2. The sample size in CPR studies can influence the generalizability of the results. Smaller studies may lack the statistical power to detect significant effects, leading to inconclusive or divergent findings. This study involved 146 participants, which is relatively robust compared to smaller studies, allowing for more reliable statistical analysis. Variations in study design also contribute to differing results. For instance, some studies may use a randomized controlled trial (RCT) design, while others may be observational or quasi-experimental. RCTs are generally more robust in establishing causal relationships but can be more challenging to implement in real-world settings, leading to differences in outcomes.

3. The level and type of training provided to participants can significantly impact their performance. Some studies might involve participants with prior CPR training, while others include completely untrained individuals. In this study, participants received a standardized briefing before performing CPR, which might have helped maximize the benefits of the feedback device.

The findings from this study support the integration of audiovisual feedback devices into CPR training programs, particularly for non-medical caregivers. Incorporating feedback devices into CPR training could become a standard practice, ensuring that trainees receive real-time feedback on their performance. This approach aligns with AHA guidelines, which emphasize the importance of high-quality chest compressions, including the correct rate and depth, as critical for successful resuscitation outcomes. Feedback devices can be especially useful in helping trainees develop muscle memory for the correct compression rate and depth, which are crucial for effective CPR.

Beyond training, these devices could be integrated into emergency response protocols, where they are used during actual CPR events. First responders and even laypersons equipped with feedback devices could potentially deliver higher-quality CPR, leading to better outcomes in out-of-hospital cardiac arrests. The AHA guidelines emphasize the importance of minimizing interruptions in chest compressions and maintaining a compression rate of 100-120 per minute. The findings from this study align with these recommendations: (1) The study demonstrated that participants using the feedback device were more likely to achieve and maintain the recommended compression rate, thus directly supporting the AHA guidelines. (2) The provision of real-time feedback helps ensure that chest compressions remain within the optimal range, reducing the likelihood of suboptimal performance, which is crucial for improving survival rates.

While the benefits of feedback devices are clear, several challenges could hinder their widespread adoption:

1. High-quality feedback devices can be expensive, potentially limiting their availability, especially in low-resource settings. The cost of purchasing, maintaining, and updating these devices could be prohibitive for some training centers or emergency response organizations.

2. Access to these devices may be limited in rural or underserved areas, where CPR training might already be infrequent. Efforts to distribute these devices more equitably will be necessary to ensure that all populations benefit from this technology.

3. Like any technological device, feedback devices require regular updates and maintenance to ensure they function correctly and remain effective. This requirement adds a layer of complexity to their use in training programs and emergency protocols.

The effectiveness of CPR training with feedback devices could vary across different cultural and regional contexts. Factors that might influence the effectiveness include the following: 1.

1. In some cultures or regions, the approach to training might be more didactic, with less emphasis on hands-on practice. Integrating feedback devices into these training environments could require significant changes to the training methodology.

2. In regions where there is a high level of comfort with technology, the adoption of feedback devices might be smoother. Conversely, in areas where technological devices are less familiar or where there is skepticism about new technologies, additional efforts might be needed to encourage their use.

3. Differences in population demographics, such as age, health status, and physical fitness, could affect how well individuals respond to CPR feedback devices. For example, older adults might find it more challenging to maintain the correct compression rate, even with feedback, compared to younger individuals.

Limitations

A significant limitation of focusing on short-term outcomes is the risk of skill decay. CPR skills, especially those requiring precision like maintaining the correct chest compression rate, tend to diminish over time without regular practice. The improvements observed in this study might not persist if participants do not receive ongoing training and reinforcement. To mitigate the risk of skill decay, the implementation of periodic refresher courses or re-assessments of CPR skills would be beneficial. These sessions could reinforce the techniques learned, ensure that participants remain proficient, and potentially introduce new or updated best practices as CPR guidelines evolve.

Performing CPR on a mannequin in a controlled environment is fundamentally different from performing CPR on a real patient during an actual cardiac arrest. In real-life situations, rescuers face emotional stress, the pressure of time, physical exhaustion, and potential distractions or interference from bystanders, which can all negatively impact CPR quality. Mannequins, while standard in CPR training, cannot fully replicate the physical characteristics of real patients. For example, real patients may have varying chest resistance, which can affect the depth and effectiveness of chest compressions. Additionally, real patients may move, vomit, or exhibit other responses that mannequins do not, further complicating the rescuer’s ability to perform high-quality CPR. Acknowledging these differences is crucial as it highlights the potential gap between simulated training outcomes and real-world performance. Therefore, while the study demonstrates the effectiveness of feedback devices in a controlled setting, the actual impact in real-life scenarios might differ, emphasizing the need for training programs that incorporate elements of real-life simulation, such as high-fidelity mannequins or scenarios that mimic the unpredictability of actual cardiac arrest situations.

Participants in this study may have had varying levels of baseline CPR knowledge, which could influence their performance. Some participants might have been more familiar with CPR techniques, while others might have been complete novices. This variability could introduce bias into the results, as those with prior knowledge might respond better to the feedback devices, skewing the overall findings.

The performance of participants was recorded, and while this was done as objectively as possible, there is always the potential for observer bias. Observers might inadvertently interpret or record performance differently based on their expectations or prior knowledge, which could affect the study’s outcomes. Automated data collection methods or blinding observers to the study’s purpose could reduce this risk. The duration and intensity of the training session before the participants performed CPR could significantly impact their performance, as the ability to understand will be different for everyone.

Summary of findings and future directions

The majority of participants (74.7%) showed a good outcome when performing chest compressions with the aid of audiovisual devices. The use of audiovisual devices, such as watches, significantly improved compression rates. Compression rates improved from 65% to 80.1% when participants used the watch. There was a statistically significant difference in compression rates with and without the watch (p-value = 0.011, kappa value = 0.167). The p-value of 0.011 indicates that there is a statistically significant difference in compression rates when using the audiovisual feedback device compared to without it. In this context, the p-value confirms that the observed improvement in chest compression quality with the device is unlikely due to random chance. This supports the conclusion that the audiovisual feedback device has a meaningful impact on CPR performance. The kappa value of 0.167 suggests only slight agreement between the chest compression rates with and without the feedback device. This low kappa value indicates that while the feedback device improves CPR performance, the consistency of this improvement across different participants is limited. Some participants responded well to the device, while others did not, suggesting variability in its effectiveness depending on individual factors such as initial skill level, experience, or ability to adapt to the feedback provided.

The study's findings could significantly influence how CPR training programs are structured. The use of audiovisual feedback devices in training could be emphasized, particularly for non-medical personnel or those with limited experience in CPR. Given the variability in device effectiveness, it might be beneficial to tailor training programs based on the participants' initial skill levels. For example, novice learners might require more guided practice with feedback devices, while experienced individuals could benefit from more advanced, scenario-based training that includes feedback. The results of this study could inform updates to CPR guidelines, such as those provided by AHA. Incorporating audiovisual feedback devices into standardized CPR training protocols could be recommended to ensure that trainees achieve the correct compression rates. Public health policies could also support the broader adoption of such devices, potentially subsidizing their cost for training centers or mandating their use in CPR certification programs.

## Conclusions

CPR training programs should consider integrating audiovisual feedback devices in a way that accounts for participant variability. For instance, introductory sessions could use the devices to help learners master basic compression techniques, while more advanced sessions could simulate real-life scenarios where learners must maintain CPR quality under stress. To address the potential for skill decay, training programs could incorporate periodic refresher courses that utilize audiovisual feedback devices. These sessions would reinforce correct compression techniques and allow participants to practice maintaining high-quality CPR over time. Future research and training should include simulations that mimic the challenges of real-life cardiac arrest situations. These could involve factors like emotional stress, physical fatigue, and environmental distractions to test how well the audiovisual devices perform under pressure.

The effectiveness of audiovisual feedback devices in real-life cardiac arrest scenarios remains an open question. Research should explore how these devices perform when rescuers are under stress or fatigued, as well as in diverse populations with varying levels of CPR experience. Longitudinal studies could be conducted to assess how well participants retain CPR skills over time when trained with audiovisual feedback devices. These studies could involve follow-up assessments at regular intervals to determine if and when skill decay occurs and how ongoing training can mitigate it.

## Figures and Tables

**Figure 1:**
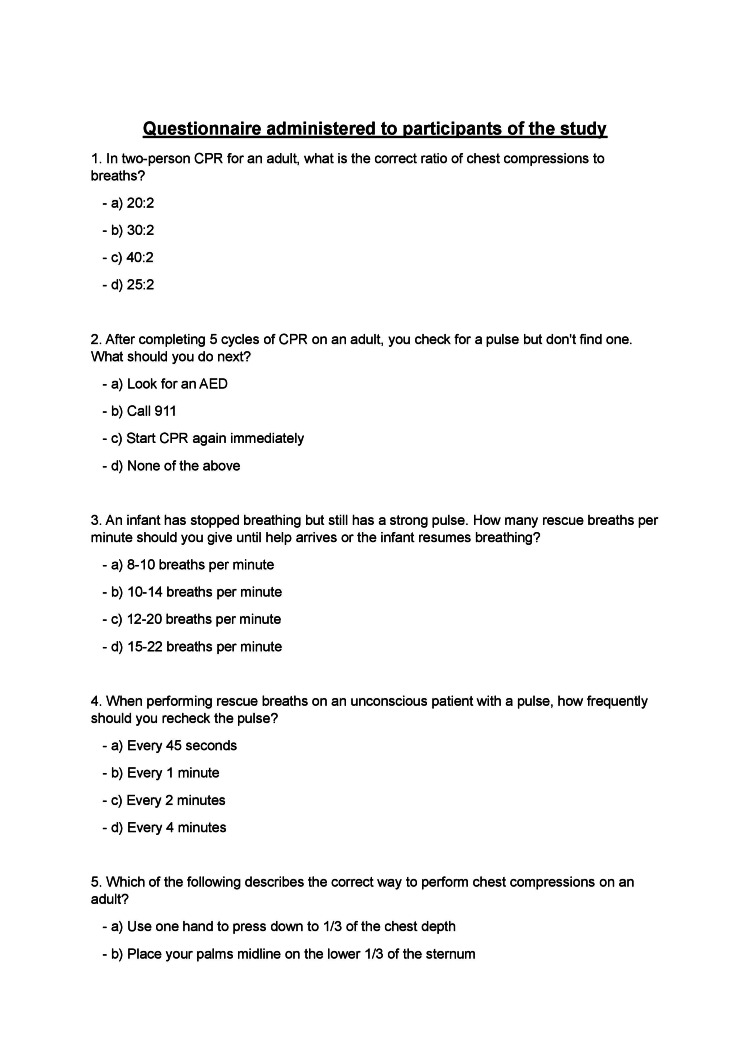
Questionnaire page 1

**Figure 2:**
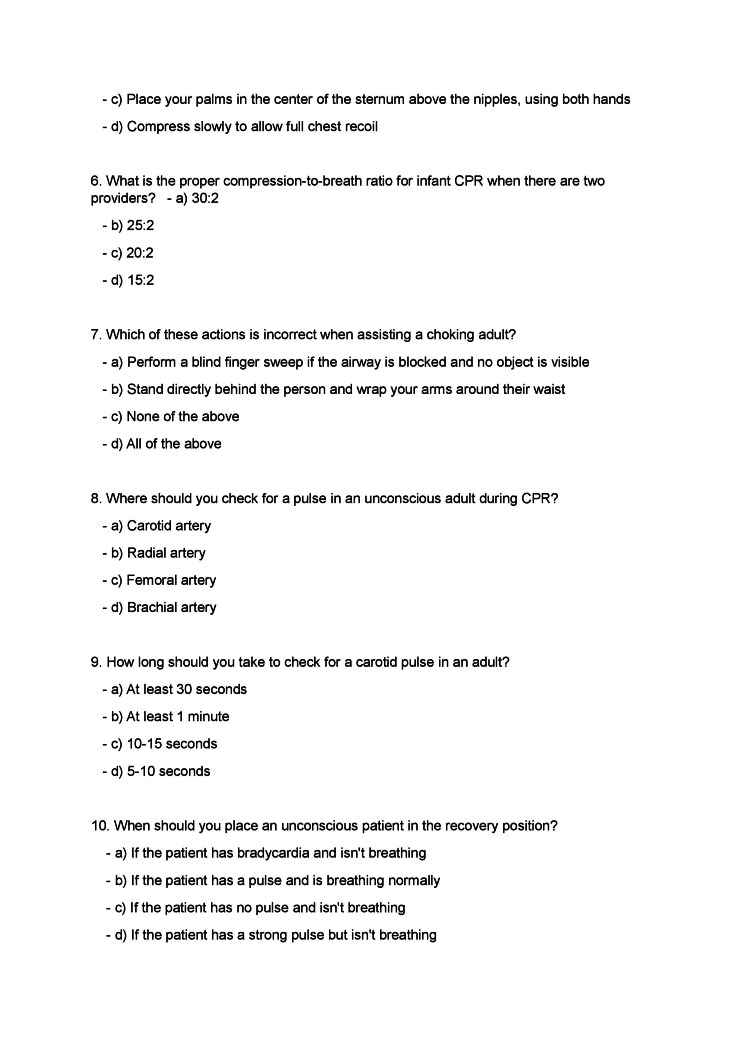
Questionnaire page 2

**Figure 3:**
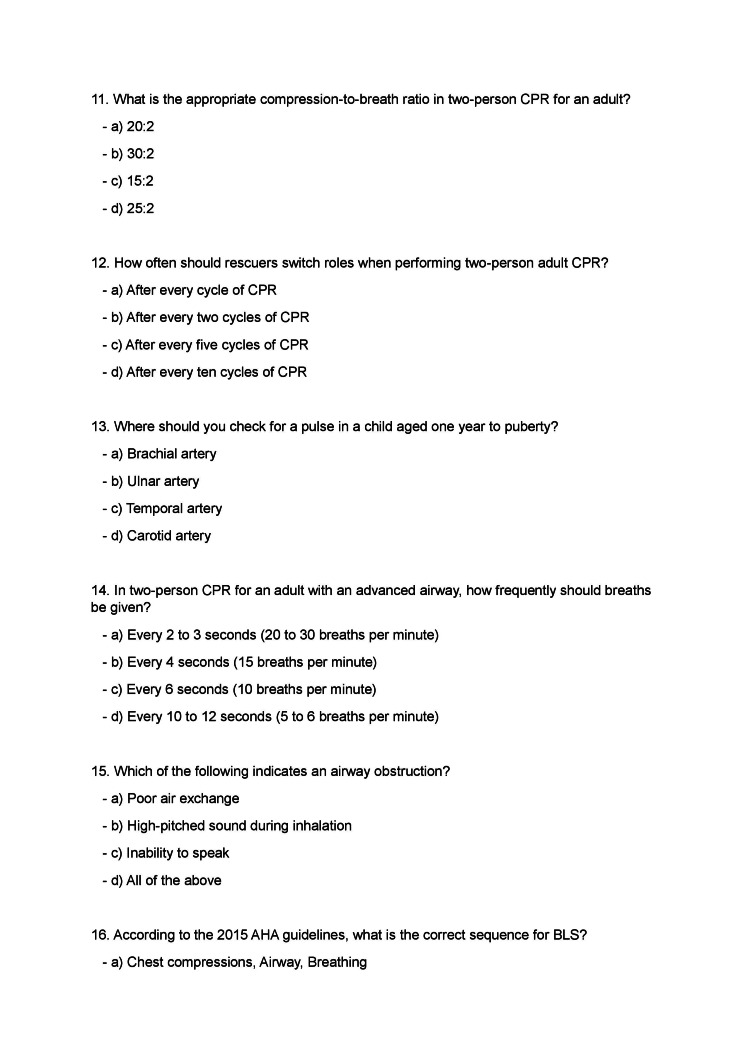
Questionnaire page 3

**Figure 4:**
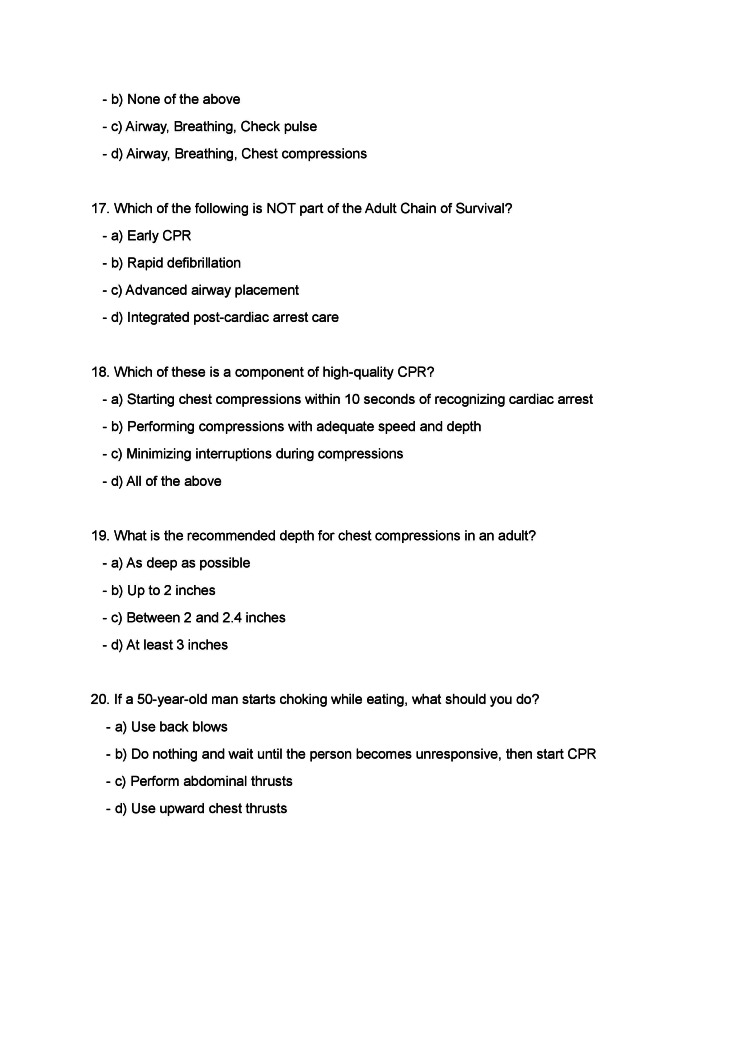
Questionnaire page 4

## Appendices

Questionnaire
